# Supplementary material for: Age, but not short-term intensive swimming, affects chondrocyte turnover in zebrafish vertebral cartilage
Source: PeerJ. 2018 Oct 1;6:e5739. doi: 10.7717/peerj.5739 (PMC6171498; doi:10.7717/peerj.5739)
Supplement: Supplemental Information 1 — Supplementary tables include raw data and experimental details. [file peerj-06-5739-s001.docx]

Table S1 A representative log file content for microCT scanning

| [System] |
| --- |
| Scanner=Skyscan1076 |
| Instrument SN=08E02052 |
| Hardware=C |
| Secure mode=OFF |
| Software=Version 3. 1 (build 0) |
| Home Directory=[...] |
| Tube=Hamamatsu 100/250 |
| Camera=Princeton Instruments |
| Camera Pixel Size (um)= 12.57 |
| Camera X/Y Ratio=1.0029 |
|  |
| [Acquisition] |
| Data Directory=[...] |
| Filename Prefix=[...] |
| Source Voltage (kV)= 80 |
| Source Current (uA)= 124 |
| Filter=Al 0.5 mm |
| Object to Source (mm)=121.000 |
| Camera to Source (mm)=165.000 |
| Number Of Files= 397 |
| Number Of Rows= 2672 |
| Number Of Columns= 4000 |
| Optical Axis (line)=1360 |
| Image Pixel Size (um)= 9.1350 |
| Image Format=TIFF |
| Depth (bits)=16 |
| Data Offset (bytes)= 264 |
| Horizontal overlap (pixel)=0 |
| Camera horizontal position=Center |
| Visual Camera=OFF |
| Screen LUT=0 |
| Exposure (ms)= 1700 |
| Rotation Step (deg)=0.500 |
| Frame Averaging=On (1) |
| Scanning position=-112.800 mm |
| Suggested beam-hardening correction=10 |
| Suggested HU-Calibration=129000 |
| Number of connected scans=1 |
| Use 360 Rotation=NO |
| Rotation Direction=CC |
| Scanning Trajectory=ROUND |
| Type Of Motion=STEP AND SHOOT |
| Camera Offset=OFF |
| Scanning Start Angle=0.000 |
| Study Date and Time=[...] |
| Scan duration=00:25:42 |
|  |
| [Reconstruction] |
| Reconstruction Program=NRecon |
| Program Version=Version: 1.6.10.2 |
| Program Home Directory=[...] |
| Reconstruction engine=GPUReconServer |
| Engine version= |
| Reconstruction from batch=No |
| Postalignment=0.00 |
| Reconstruction servers= [...] |
| Dataset Origin=Skyscan1076 |
| Dataset Prefix=[...] |
| Dataset Directory=[...] |
| Output Directory=[...] |
| Time and Date=[...] |
| First Section=2 |
| Last Section=2667 |
| Section to Section Step=1 |
| Sections Count=2666 |
| Result File Type=BMP |
| Result File Header Length (bytes)=1134 |
| Result Image Width (pixels)=1276 |
| Result Image Height (pixels)=1276 |
| Pixel Size (um)=9.13668 |
| Reconstruction Angular Range (deg)=198.50 |
| Use 180+=OFF |
| Angular Step (deg)=0.5000 |
| Smoothing=2 |
| Smoothing kernel=0 (Asymmetrical boxcar) |
| Ring Artifact Correction=5 |
| Draw Scales=OFF |
| Object Bigger than FOV=OFF |
| Reconstruction from ROI=ON |
| ROI Top (pixels)=2733 |
| ROI Bottom (pixels)=1455 |
| ROI Left (pixels)=1409 |
| ROI Right (pixels)=2687 |
| ROI reference length=4000 |
| Filter cutoff relative to Nyquist frequency=100 |
| Filter type=0 |
| Filter type description=Hamming (Alpha=0.54) |
| Undersampling factor=1 |
| Threshold for defect pixel mask (%)=3 |
| Beam Hardening Correction (%)=10 |
| CS Static Rotation (deg)=0.00 |
| Minimum for CS to Image Conversion=0.000000 |
| Maximum for CS to Image Conversion=0.030000 |
| HU Calibration=OFF |
| Cone-beam Angle Horiz.(deg)=17.175762 |
| Cone-beam Angle Vert.(deg)=11.521132 |

All log entries are listed, but the values of entries that are irrelevant to the scanning are replaced with “[…]”.

Table S2 Body lengths (cm) of zebrafish in Figure 1C-D

| 4-mo | 12-mo | Contorl | | Exercise | |
| --- | --- | --- | --- | --- | --- |
|  |  | +0d | +14d | +0d | +14d |
| 2.86 | 2.969 | 3.24 | 3.24 | 3.44 | 3.39 |
| 2.75 | 3.077 | 3.31 | 3.39 | 3.55 | 3.42 |
| 2.87 | 3.25 | 3.29 | 3.45 | 3.49 | 3.34 |
| 2.49 | 3.199 | 3.31 | 3.34 | 3.63 | 3.58 |
| 2.47 | 3.204 | 3.45 | 3.3 | 3.7 | 3.55 |
| 2.7 | 2.97 | 3.46 | 3.5 | 3.4 | 3.28 |
| 2.55 | 3.093 | 3.01 | 3.4 | 3.47 | 3.45 |
| 2.53 | 3.019 | 3.28 | 3.08 | 3.2 | 3.18 |
|  | 2.951 | 3.24 | 3.32 |  |  |
|  | 3.185 |  |  |  |  |
|  | 3.309 |  |  |  |  |
|  | 3.198 |  |  |  |  |

Table S3 Body weight (g) of zebrafish in Figure 1E-F

| 4-mo | 12-mo | Contorl | | Exercise | |
| --- | --- | --- | --- | --- | --- |
|  |  | +0d | +14d | +0d | +14d |
| 0.2 | 0.186 | 0.24 | 0.28 | 0.38 | 0.33 |
| 0.18 | 0.239 | 0.33 | 0.36 | 0.32 | 0.28 |
| 0.19 | 0.267 | 0.34 | 0.36 | 0.39 | 0.37 |
| 0.14 | 0.269 | 0.3 | 0.34 | 0.38 | 0.4 |
| 0.13 | 0.258 | 0.32 | 0.33 | 0.45 | 0.44 |
| 0.16 | 0.194 | 0.29 | 0.33 | 0.31 | 0.29 |
| 0.15 | 0.225 | 0.27 | 0.33 | 0.37 | 0.35 |
| 0.15 | 0.202 | 0.3 | 0.29 | 0.29 | 0.26 |
|  | 0.185 | 0.3 | 0.32 |  |  |
|  | 0.24 |  |  |  |  |
|  | 0.258 |  |  |  |  |
|  | 0.259 |  |  |  |  |

Table S4 Bone mineral density of zebrafish in Figure 2C-D

| 12-mo | | | |
| --- | --- | --- | --- |
| Ctrl-D0 | Ctrl-D14 | Exer-D0 | Exer-D14 |
| 0.34591 | 0.34892 | 0.39737 | 0.40912 |
| 0.34864 | 0.35129 | 0.37968 | 0.39962 |
| 0.35101 | 0.36261 | 0.40146 | 0.41698 |
| 0.34753 | 0.37122 | 0.37578 | 0.38868 |
| 0.3527 | 0.36876 | 0.39999 | 0.39899 |
| 0.36265 | 0.37083 | 0.37367 | 0.3643 |
| 0.35287 | 0.37624 | 0.3704 | 0.3787 |
| 0.35767 | 0.36167 | 0.37833 | 0.38177 |

Table S5 Area size (µm^2^) of Type II collagen in zebrafish 4^th^ vertebrate in Figure 4D and F

| 4-mo | 12-mo | Ctrl+D14 | Exer+D14 |
| --- | --- | --- | --- |
| 8822.326 | 16734.66 | 7087.44 | 17920.34 |
| 9128.762 | 14520.72 | 12970.58 | 16723.56 |
| 7010.498 | 18531.07 | 7924.919 | 12671.05 |
| 8421.326 | 14680.48 | 14600.79 | 12443.81 |
| 8849.64 | 13927.99 | 14715.39 | 15587.84 |
| 5798.035 | 6226.311 | 10702.63 | 14493.52 |
| 6108.78 | 16665.04 |  | 14829.83 |

Table S6 Intensity density (/µm^2^) of Type II collagen in zebrafish 4^th^ vertebrate in Figure 4E and G

| 4-mo | 12-mo | Ctrl+D14 | Exer+D14 |
| --- | --- | --- | --- |
| 80554.43 | 113315 | 108142.8 | 133505.5 |
| 71194.2 | 127705.4 | 89241.64 | 110423.4 |
| 71247.15 | 126399.8 | 80024.34 | 100095.4 |
| 76017.49 | 144318.7 | 127043.3 | 92868.75 |
| 98978.09 | 117662 | 113558.9 | 103841 |
| 62457.98 | 124817 | 106880.1 | 118569.3 |
| 56937.86 | 153087.5 |  | 92572.71 |

Table S7 Area size (µm^2^) of Safranin O in zebrafish 4^th^ vertebrate in Figure 5D

| 4-mo | 12-mo | Ctrl+D14 | Exer+D14 |
| --- | --- | --- | --- |
| 28469.58 | 42189.82 | 39331.06 | 47269.96 |
| 33394.13 | 43256.89 | 44050.11 | 49331.62 |
| 26856.92 | 62168.17 | 51673.14 | 55725.99 |
| 27244.64 | 45537.3 | 46537.45 | 52936.9 |
| 27105.25 | 32756.74 | 48111.36 | 54886.94 |
| 24758.11 | 53883.51 | 45485.44 | 44110.23 |
| 28692.59 | 44440.6 | 50125.09 | 50105.6 |
| 27723.62 | 55289.73 | 46496.15 | 44575.35 |

Table S8 Intensity density (/µm^2^) of Safranin O in zebrafish 4^th^ vertebrate in Figure 5E

| 4-mo | 12-mo | Ctrl+D14 | Exer+D14 |
| --- | --- | --- | --- |
| 1900.333 | 1739.911 | 2043.863 | 2117.903 |
| 2114.056 | 1419.391 | 1992.418 | 2198.989 |
| 2046.195 | 1774.717 | 2401.871 | 2300.248 |
| 1701.522 | 1770.412 | 2214.85 | 2350.923 |
| 1671.594 | 1770.932 | 2364.333 | 2232.921 |
| 1757.407 | 1683.74 | 2166.915 | 2168.128 |
| 2072.085 | 1816.484 | 2151.834 | 2574.987 |
| 1802.081 | 1933.637 | 1975.847 | 2333.491 |

Table S9 Cell counts in Safranin O stained area in zebrafish 4^th^ vertebrate in Figure 5F and G

| 4-mo | 12-mo | Ctrl+D14 | Exer+D14 |
| --- | --- | --- | --- |
| 1056 | 1335 | 1221 | 1308 |
| 1241 | 1578 | 1159 | 1547 |
| 868 | 1381 | 1411 | 1432 |
| 964 | 1537 | 1521 | 1382 |
| 985 | 1268 | 1181 | 1331 |
| 1150 | 1691 | 1075 | 1241 |
| 1053 | 1326 | 1302 | 1469 |
| 1095 | 1318 | 1428 | 1212 |

Table S10 DAPI and TUNEL positive counts in zebrafish 4^th^ vertebrate in Figure 6A and B

| 4-mo | | 12-mo | | Ctrl+D14 | | Exer+D14 | |
| --- | --- | --- | --- | --- | --- | --- | --- |
| TUNEL | DAPI | TUNEL | DAPI | TUNEL | DAPI | TUNEL | DAPI |
| 126 | 1330 | 566 | 2091 | 255 | 1697 | 367 | 1681 |
| 154 | 1519 | 429 | 1796 | 343 | 1684 | 330 | 1861 |
| 60 | 1239 | 551 | 2616 | 229 | 1968 | 440 | 2020 |
| 127 | 1356 | 420 | 1890 | 483 | 2092 | 288 | 1696 |
| 154 | 1473 | 147 | 1494 | 348 | 1769 | 390 | 1779 |
| 212 | 1728 | 626 | 2110 | 344 | 1784 | 265 | 1579 |
| 87 | 1144 | 470 | 1671 | 442 | 1930 | 323 | 1754 |
|  |  | 598 | 1696 |  |  | 325 | 1608 |

Table S11 DAPI and BrdU positive counts in zebrafish 4^th^ vertebrate in Figure 6C and D

| 4-mo | | 12-mo | | Ctrl+D14 | | Exer+D14 | |
| --- | --- | --- | --- | --- | --- | --- | --- |
| BrdU | DAPI | BrdU | DAPI | BrdU | DAPI | BrdU | DAPI |
| 3 | 1077 | 3 | 1038 | 1 | 1214 | 0 | 1143 |
| 117 | 1426 | 1 | 1114 | 0 | 1134 | 0 | 1303 |
| 7 | 1363 | 0 | 1179 | 0 | 1460 | 1 | 1251 |
| 18 | 1104 | 0 | 1352 | 2 | 1582 | 2 | 1501 |
| 8 | 935 | 2 | 1133 | 0 | 1080 | 0 | 1340 |
| 6 | 1181 | 2 | 1083 | 1 | 1115 | 0 | 1230 |
| 27 | 1159 | 1 | 1369 | 1 | 1438 | 0 | 1344 |
| 128 | 1104 | 2 | 1389 | 1 | 1338 | 2 | 1342 |
|  |  | 0 | 1013 |  |  |  |  |
|  |  | 2 | 840 |  |  |  |  |
|  |  | 1 | 1084 |  |  |  |  |

Table S12 BrdU pulse and chase in zebrafish 4^th^ vertebrate in Figure 6E

| 4mo+15d | | 4mo+30d | | 12mo+15d | |
| --- | --- | --- | --- | --- | --- |
| BrdU | DAPI | BrdU | DAPI | BrdU | DAPI |
| 25 | 1101 | 106 | 1326 | 0 | N/A |
| 7 | 1207 | 17 | 1274 | 0 | N/A |
| 4 | 950 | 0 | 986 | 0 | N/A |
| 1 | 944 | 0 | 967 | 0 | N/A |
| 0 | 1130 | 7 | 1105 | 0 | N/A |
| 1 | 1080 | 2 | 1269 | 0 | N/A |

N/A: not analyzed

Table S13 Estimated curvature of the spines in intensive exercised zebrafish

|  | Before | After |
| --- | --- | --- |
| #4 | 19.16235674 | 12.2742165 |
| #5 | 5.087616985 | 20.55535654 |
| #8 | 2.162313433 | 8.562380552 |
| #10 | 11.76304828 | 8.220812997 |
| #13 | 30.57472308 | 10.54808687 |
| #15 | 19.45558875 | 5.532300562 |
| #16 | 10.42552002 | 6.088527222 |
| #17 | 2.014999093 | 36.5446392 |

The curvature of the spine was estimated by the average deviating distance from a straight line between fifth to thirty-first vertebrae. Simply, cross-section area between fifth to thirty-first vertebrae was divided by its straight length.
