# Supplementary figures and images for: Age, but not short-term intensive swimming, affects chondrocyte turnover in zebrafish vertebral cartilage
Source: PeerJ. 2018 Oct 1;6:e5739. doi: 10.7717/peerj.5739 (PMC6171498; doi:10.7717/peerj.5739)

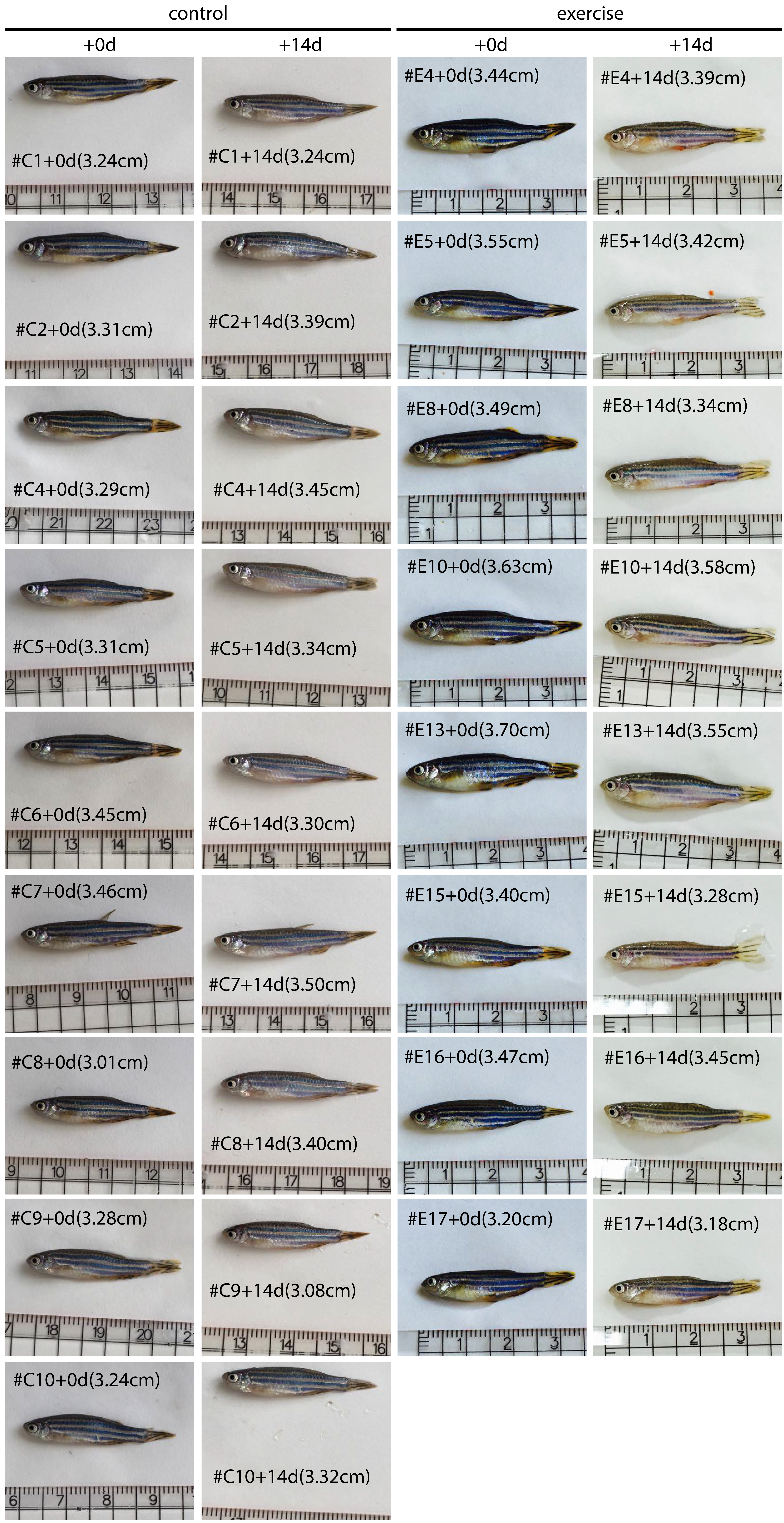

Supplement: Supplemental Information 3 — Body lengths of 12-month-old zebrafish were measured in micrographs with ImageJ before (+0d) and after (+14d) a 14-day intensive exercise training. [file peerj-06-5739-s003.png]
